# Oral health condition and occurrence of depression in the elderly

**DOI:** 10.1097/MD.0000000000012490

**Published:** 2018-10-12

**Authors:** Katarzyna Skośkiewicz-Malinowska, Barbara Malicka, Marek Ziętek, Urszula Kaczmarek

**Affiliations:** a Department of Conservative Dentistry and Pedodontics; b Department of Periodontology, Wroclaw Medical University, Poland.

**Keywords:** depression, elderly, oral health, PHQ-9

## Abstract

Depression is a common disorder among the elderly; however, it is not a standard element of the ageing process. Depression can affect oral health as a result of neglecting oral hygiene procedures, cariogenic nutrition, avoidance of necessary dental care which leads to an increased risk of dental caries and periodontal disease.

Assessment of the relationship of oral health parameters with depression.

500 subjects aged ≥65 (mean 74.4 ± 7.4) were involved in the study. Dental condition (decay-missing-filled index [DMFT], number of missing teeth [MT], removable denture wearing, teeth mobility), periodontal condition (bleeding on probing [BoP], pocket depth [PD], loss of attachment), oral dryness (the Challacombe Scale) and depression according to the Patient Health Questionnaire-9 (PHQ-9) scale were assessed.

Depression on a minimal level was detected in 60.2% of the subjects, mild—in 22.2%, moderate—in 6.0% and moderately—in 2.6%. The mean of the PHQ-9 scale was 3.56 ± 4.07. Regression analysis showed a positive relationship of the PHQ-9 value with DMFT, the number of MT, oral dryness and with age. No correlation was observed between other examined oral health indicators, such as periodontal condition (BoP, PD, clinical attachment level), periodontitis, removable denture wearing a PHQ-9.

The results of our study have shown that among people aged 65 and over, the severity of depression increases with a higher number of MT, the number of decayed teeth, as well as prevalence of oral dryness.

## 1 Introduction

Depression is a common disorder among the elderly; however, it is not a standard element of the ageing process. On the contrary, it is observed that most elderly people are satisfied with life despite diseases and physical problems. As people get older, they experience new situations and circumstances as a result of which they need to face, for example, reduced or lack of vocational activity, loss of a life partner, the need to overcome physical barriers on a daily basis, as well as emotional barriers related to loneliness and the transience of life. Problems related to insomnia, loss of appetite, as well as to broadly defined functioning in the society may negatively influence a person's well-being. An intensified, lasting at least 2 weeks, feeling of sadness and low spirits which impact everyday functioning are diagnosed as depression which demonstrates itself through loss of interest in activities which were previously considered enjoyable and through difficulties with undertaking them.^[1]^

Depression is a common and serious medical illness that negatively affects how you feel, the way you think and how you act.^[2]^ Furthermore, depression plays a very important role in chronic pain syndromes, because it has the potential to intensify perceived pain and reduce an individual's capacity to tolerate pain.^[3,4]^ International research among community-dwelling older adults, involving also the Polish population, demonstrated a correlation with depression and lower cognitive function and severe disability in the elderly.^[5]^ In Poland, around 10% of the population suffer from depression;^[6]^ however, depression disorders which do not fulfil the diagnostic criteria of the disease are identified in ca. 15% men and 25% women.^[7]^ Previous research conducted in Poland and aiming at assessing the prevalence of depression among the elderly indicates more than 25% symptoms of depression of the examined population.^[8]^ Moreover, The PolSenior project which is a multicenter, publicly funded research project commissioned by the Ministry of Science and Higher Education, and the aim of which is the assessment of the health and social status of elderly subjects in Poland, reported that the morbidity of depressive disorders increased with age (20% in the 55–59 age group, 25% in the 65–79 age group, 33% in those 80 and over).^[9]^

Reduced energy and motivation associated with depression can affect oral health by neglecting oral hygiene procedures, which leads to an increased risk of dental caries and periodontal disease, cariogenic nutrition, avoidance of necessary dental care and antidepressant-induced xerostomia. According to research conducted by Park et al, more frequent incidents of toothache were reported by patients with depression, they also frequently reported uncomfortable mastication, as well as temporomandibular joint symptoms and periodontal bleeding.^[10]^ Chronic diseases such as dental caries are still highly prevalent in older adults, and the risk of tooth loss in old age is high. Caries and periodontal disease are progressive processes which lead to tooth loss if not treated adequately. Tooth loss will presumably cause functional impairment, for example, with regard to chewing and esthetics, depending on the location of tooth loss, which might ultimately affect health-related quality of life (HRQoL).^[10]^

A reciprocal relationship between poor oral condition and mental health was noted; however, in most cases, mental condition was assessed with the use of comprehensive psychometric tools utilized to determine HRQoL which also consists of mental health. This research revealed that tooth loss and dental pain impaired HRQoL.^[11,12]^ Loss of teeth leads to uncomfortable chewing and speaking problems, poor aesthetics limiting social activities and chronic intra oral pain (tooth, periodontal, or denture pain).^[13–15]^

The aim of the study was to determine the relationship between oral health and depression.

## 2 Materials and methods

### 2.1 Investigation population

The study was being conducted for 24 months (from January 2015 to December 2016), after obtaining a consent of the Bioethics Committee of Wroclaw Medical University KB 420/2015. The study recruited 500 subjects of both genders (180 men and 320 women) at the age of ≥65 (mean age 74.4 ± 7.4, age range 65–99) who were urban citizens of Wroclaw. The participation in the survey was voluntary.

Data on the total number of 65 and over subjects living in Wroclaw were derived from the Central Statistical Office (Demographic Yearbook of Poland 2015). A minimum sample size was calculated based on data concerning caries prevalence in this age group with the assumed significance level of 95% and with ± 5% error tolerance. It turned out to be 383; therefore, the number of the examined subjects exceeded this figure.

### 2.2 Survey inclusion/exclusion criteria

The inclusion criteria for the study were: age of 65 and over, place of living (local resident), able to communicate, and a written consent to participate in the survey. The exclusion criteria were some coexisting systemic diseases in which dental pocket probing leading to transient bacteremia might have posed a risk for the patient's overall health condition, as well as lack of a written consent, or mental disorders, which would have made filling out the questionnaire impossible. Systemic diseases which were determines as exclusion criteria were: cardiovascular diseases (patients with heart valves, after heart transplant, with congenital heart diseases, or with infective endocarditis), blood diseases (thrombocytopenia, hemophilia, von Willebrand disease), viral diseases (B and C type hepatitis, AIDS/HIV), as well as patients with Multi-Drug Resistant Organisms (MDRO).

The participation in the survey was voluntary. The patients were enrolled based on information provided in press, on the radio and on the Internet, as well as in Senior Citizens Associations (Klub Seniora), at the University of the Third Age (Uniwersytet Trzeciego Wieku), church organizations, primary care outpatient clinics, pharmacies, and residential homes.

### 2.3 Sociomedical examination

The demographic and personal information and medical history were obtained from the subjects. Before the appointment, the patients were asked to provide written information about current and past diseases, as well as medications currently taken by them. The data included the date of birth and gender, marital status, level of education, and monthly income. Medical data comprised type of past and present diseases which were categorized according to the systems.

### 2.4 Clinical dental examinations

The examinations were carried out by 2 examiners who had been calibrated beforehand. The calibration consisted of examining 10 patients twice with intervals of at least 30 minutes between the examinations. The patients involved in the calibration examinations represented a full range of conditions assessed in the planned examination. The results of the examinations were compared between the examiners, and the results of 2 examinations conducted by the same examiner were analyzed too. Any deviations were discussed with an expert. Inter-examiner reliability and intra-examiner reliability was assessed with the use of the Cohen's kappa coefficient. The range of the values of this coefficient is <−1; 1. Values within the range from −1 to 0 indicate lower assessment compatibility in comparison with random compatibility; in the case of positive values (from 0 to 1), the closer they are to 1, and the more compatible they are. Cohen's kappa coefficient values are interpreted in the following way: >0.8—good compatibility, 0.6–0.8—significant compatibility, and 0.4–0.6—moderate compatibility. In own research, Cohen's kappa coefficient values were 0.874 and 0.870 (between the examiners and for the same examiner respectively), which indicates good compatibility.

The following were examined: dental condition based on the World Health Organization (WHO) criteria (the decay-missing-filled index [DMFT] and components),^[16]^ periodontal condition (bleeding on probing [BoP], pocket depth [PD], clinical attachment level [CAL]),^[16]^ severe periodontitis according to Page and Eke's definition,^[17]^ teeth mobility according to Miller, removable denture wearing, oral dryness according to Challamcobe's clinical indicator (Clinical Oral Dryness Score [CODS]).^[18]^ The clinical examination was conducted with the use of artificial light and the WHO 621 probe.

The DMFT index was calculated as a sum of its components: DT—number of decayed teeth, MT—number of missing teeth and FT—number of filled teeth.

The periodontum was examined around all natural teeth with the use of the WHO 621 probe at 6 measurement points around the tooth (disto-, medio-, mesio-vestibular, and mesio-, medio-, disto-oral) and the most severe condition was recorded for a given tooth. Patients with fewer than 2 natural teeth were excluded from the periodontal examination.

The following items were assesses:

1. BoP (the occurrence of bleeding during the probing or within 10 to 30 seconds following the probing; expressed as the percentage of bleeding that occurs at one of the measurement points at least);
2. PD in mm, calculated at 6 points for every tooth as the distance between the bottom of the pocket and the gingival margin;
3. CAL in mm, calculated as a sum of recession and PD;
4. Tooth mobility was recorded per tooth. Tooth mobility was measured without a measuring device. The tooth crown was simply moved between the fingertip and a rigid instrument in labio-oral direction. That is, with the patient's mouth open, each tooth is grasped between fingertip and instrument handle and is moved in labio-oral direction. The mobility was scored from 0 to 3.

The periodontal condition was categorized according to Page and Eke's definition^[17]^ into:

- mild periodontitis—2 or more interproximal sites with CAL≥3 mm on and 2 or more interproximal sites with PD≥4 mm,
- moderate periodontitis—2 or more interproximal sites with CAL≥4 mm or 2 or more interproximal sites with PD≥5 mm,
- severe periodontitis—2 or more interproximal sites with CAL≥6 mm and 1 or more interproximal sites with PD≥5 mm.

The dental prosthetic status was assessed with regards to the presence of partial removable dentures (tissue-borne and frame dentures), as well as full dentures (with mucosal support and overdentures) and unreplaced teeth.

Oral dryness was determined according to the Challacombe Scale which serves as the CODS.^[18]^ which is based on 10 characteristic symptoms related to saliva deficiency in the oral cavity:

(1) mirror sticks to oral mucosa;
(2) mirror sticks to tongue;
(3) saliva frothy;
(4) no saliva pooling in floor of mouth;
(5) tongue shows generalized shortened papillae (mild depapillation);
(6) altered gingival architecture (i.e., smooth);
(7) glassy appearance of oral mucosa, especially palate;
(8) tongue lobulated/fissured;
(9) cervical caries (more than 2 teeth);
(10) debris on palate or sticking to teeth.

The extent of dryness was categorized based on the number of observed symptoms as mild (1–3), moderate (4–6), or severe.^[18]^

### 2.5 Psychometric examination

Depression was determined with the use of the Patient Health Questionnaire-9 (PHQ-9), as it is a tool which can be used by doctors of specialties other than psychiatry to screen patients for depression. Questionnaire was filled out by the patients themselves after they had been provided with the instructions.^[19]^ The answers to the questions related to well-being. in the past 2 weeks were categorized according to the frequency of occurrence on a scale from 0 to 3 (0- not at all, 1—several days, 2—more than half the day, 3—nearly every day). The total number of scores determined the severity of depression: 0–4 lack of or minimal symptoms, 5 to 9 mild symptoms, 10 to 14 moderate symptoms, 15–19 moderately severe symptoms, 20 to 27 severe symptoms.^[19]^

### 2.6 Statistical analysis

For quantitative variables, the normality of empirical distributions was verified using the Kolmogorov–Smirnov test. Subsequently, mean values and standard deviations were calculated, and mean values were compared between the 2 patient groups in Student *t* test.

Discreet and ordinal variables were grouped in contingency tables, with numbers (n) and fractions (%) calculated. The independence of qualitative characteristics was verified using Pearson's chi-squared test. The strength of correlations was determined by calculating Pearson correlation coefficients (r) and significance level (p).

The association of the independent variable (PHQ-9) was tested in multiple linear regression analysis using the forward stepwise method. For independent variables, standardized coefficients and regression coefficients beta with 95% CI were calculated. The results of all the tests were considered significant at *P* value < .05.

Calculations were performed using the STATISTICA v. 12 software suite (StatSoft, Cracow, Poland).

## 3 Results

### 3.1 The demographic and personal information

The study involved 500 inhabitants of Wroclaw of both genders aged 65 and over. The mean age of the subjects was 74.4. Women constituted 64.0% of the overall number of subjects. Most of the subjects lived with their family member/s (62.0%) and they did not require any help with completing everyday activities (90.8%) (Table 1). Among the subjects, 51.0% completed education on the secondary level, 32.8% on the higher level. 45.2% of subjects reported average income per household.

**Table 1:**
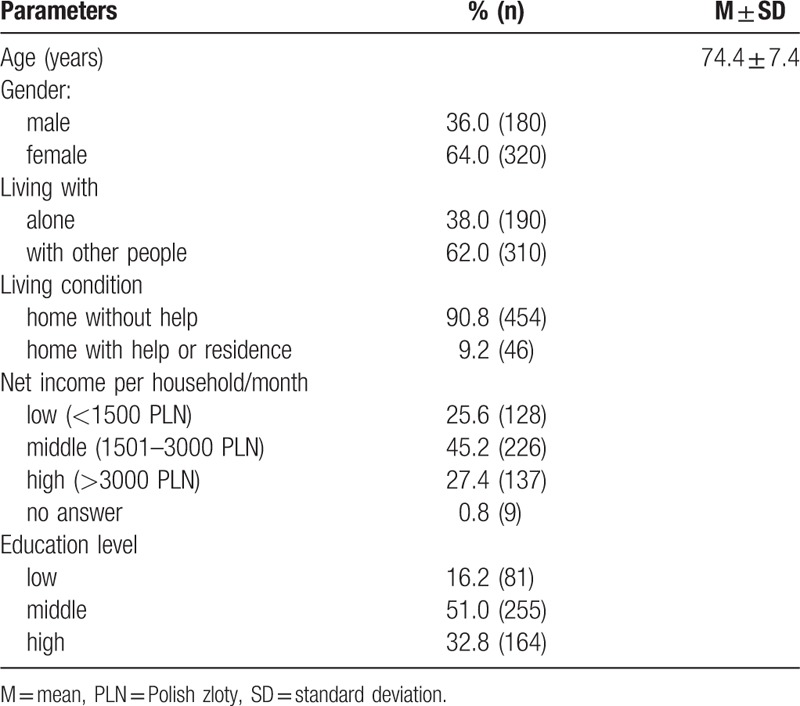
Sociodemographic characteristic of the subjects.

### 3.2 General health condition

The past and current disorders reported by the subjects were related to all systems and organs; however, most frequently, they were related to cardiovascular diseases and diabetes (Table 2). The subjects relatively often suffered from 2 (27.0%) or 3 (18.8%) coexisting diseases.

**Table 2:**
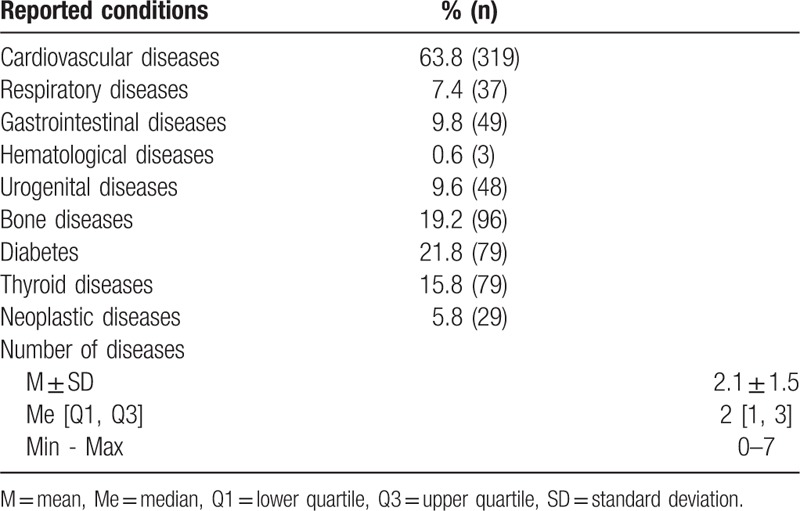
Medical condition of the subjects.

### 3.3 Oral health condition

Dental caries affected all of the subjects. The subjects revealed the mean value of DMFT 27.5 ± 5.0, out of which DT = 1.5 ± 2.7, MT = 19.0 ± 9.6, FT = 7.0 ± 6.3. The prevalence of moderate and severe periodontitis in the subjects having at least 2 natural teeth was 39.3% and 50.3%, respectively. The percentages of the sites with BoP were 55.0% ± 43.2, pocket ≥4 mm 50.2% ± 26.6 (mean 3.7 ± 0.9 mm), loss of attachment 26.1% ± 27.1 (4.3 ± 1.4 mm). 5.3% of mobile teeth ≥1 degree was found. The partial removable denture in the maxilla was worn by 30.8% subjects and in the mandible by 20.0%; full removable denture was worn by 25.0% and 22.6% respectively. Oral dryness was found in 32.8% subjects, out of whom mild was determined in 29.6%, moderate in 3.0% and severe in 0.2%.

### 3.4 Occurrence of depression 

No depression or depression on a minimal level was detected in 69.2% of the subjects (346/500), mild—in 22.2% (111/500), moderate—in 6.0% (30/500) and medium severe—in 2.6% (13/500). The mean of the PHQ-9 scale was 3.56 ± 4.07 (Table 3).

**Table 3:**
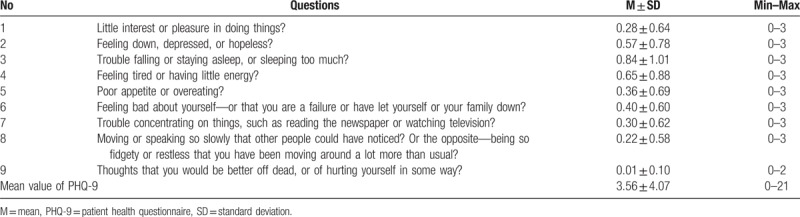
Mean values of Patient Health Questionnaire-9 scale.

### 3.5 The relationship between the PHQ-9 score with oral health parameters

Regression analysis showed a positive relationship of the PHQ-9 score with DMFT, the number of MT, oral dryness, and with age (Table 4). Based on single- and multiple-factor regression analysis gender, number of extracted teeth and number of diseases turned out as variables which were independent predictors of depression severity determined with the PHQ-9 scale. The PHQ-9 score increased with the age of the subjects; however, male subjects were the determinant of lower values. Furthermore, it increased with the rise of DMFT and the number of MT, as well as with the occurrence of oral dryness. However, there was no association observed among other examined oral health indicators, such as periodontal condition (BoP, PD, CAL), periodontitis, removable denture wearing a PHQ-9.

**Table 4:**
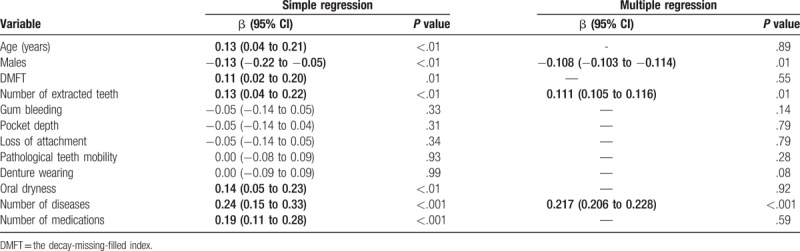
Simple and multiple regression analysis result for the variables studied against PHQ-9 scores.

In the present study, apart from the associations observed between oral health indicators and depression, the influence of the number of coexisting diseases and the number of medications taken on the frequency of depression was demonstrated.

## 4 Discussion

Depression is a common mental disorder which is characterized by lasting, for at least 2 weeks, sadness and loss of interest in activities which were considered enjoyable before, combined with an inability to perform everyday activities and tasks, to eat properly and to sleep.^[1]^ By disturbing normal life to such an extent, it also negatively impacts oral health-related self-care behaviors, which means that it may have a detrimental effect on oral health. Depression in women develops 50% more frequently than in men,^[20]^ and it is also more often observed in younger adults than in older adults.^[21]^ The occurrence of depression is conditioned by many factors, particularly by coexisting diseases. Depression determined by health condition is referred to as somatogenic depression.^[22]^ Major depression is observed in 20% to 25% of people suffering from cardiovascular diseases,^[23]^ the same percentage is also related to patients after a stroke.^[24]^ Major depression was also detected in 15% of patients with diagnosed type 2 diabetes^[25]^ and in people suffering from Alzheimer's and Parkinson's disease.^[26]^ Based on research conducted among Korean Community-Dwelling Elderly, Kim at al determined that the perceived health status was the most powerful predictor of elderly depression.^[27]^ It is known that elderly people more frequently than younger people suffer from chronic somatic disorders causing afflictions which hinder daily functioning that leads to the development of depression. Annual incidence of depression among elderly population is estimated at 15%, however, the percentage doubles after the age of 70. Taking medications, such as interferon, steroids, and benzodiazepines, is also a predisposing factor of depression.^[28,29]^ The occurrence of depression is related to an increase in disease incidence, taking medications and undergoing treatment, reduced physical activity, and deteriorated quality of life.^[30–34]^

One of the methods of diagnosing the disorder is based on psychometric tests. They are tools which are validated in advance, and which can be administered not only by psychiatrists and psychologists but also by doctors of other specialties due to lack of necessity to interpret the results. One of such tools which is used to diagnose depression among the elderly is the PHQ-9. This tool, commonly used worldwide with elderly people, is also useful to monitor treatment.^[35]^ This questionnaire screens for the presence as well as the severity of depression, and it is a diagnostic version of PRIME-MD PHQ which is used to detect common mental disorders.^[19]^

In our study, depression was detected and its severity was determined with the use of the PHQ-9. It is assumed that the Polish version of the scale is a good screening instrument to detect depression among the elderly.^[36]^ The mean score of the scale was 3.56 ± 4.07, which categorizes the subjects to a group with mild depression. Most often, the subjects reported problems with sleep (0.84 ± 1.01). Among women, the value of the scale was considerably higher than among men, which is conclusive with the results of other research.^[20]^ According to national research conducted in the USA among subjects over 18, there was prevalence of various severity of depression determined based on PHQ-9 in 25.52% women and 17.53% men.^[37]^

In our study, the PHQ-9 values significantly increased with the number of caries-affected teeth (DMFT), the number of MT and oral dryness, as well as an increased number of coexisting systemic diseases and medications taken. They are conclusive with the results of previous studies and our data also indicate the relationship between poor oral health and the development of depression.^[13,38–41]^ Yang et al in well characterized, nationally representative, population-based study in the Korean adult population found that tooth loss and dental pain had social and psychological impacts that could reduce HRQOL.^[11]^ The tooth loss of 8 to 28 teeth with dental pain group showed the highest level of impaired HRQOL in all dimension of EuroQol- 5 Dimension including anxiety/depression. It results from the fact that the loss of natural teeth can compromise normal chewing, speaking, laughing, appearance, self-image, and social interaction.^[11]^ The loss of natural teeth may have influence on prevalence of depression as well as some studies have demonstrated the impact of tooth loss on HRQoL and oral health-related quality of life.^[11,12,42–45]^

Okoro et al,^[40]^ similarly to our study, observed a relationship between depression and the number of MT. Chou at al.,^[41]^ in turn, determined the prevalence of depression (with the use of PHQ-9) among patients who came from 7 different countries (Argentina, Chile, Denmark, India, Japan, Great Britain, and USA) and who suffered from Sjögren syndrome which is characterized by dry eye and oral dryness. Subjects from all the countries revealed a significant relationship between symptoms of dryness and mild and severe depression. Therefore, the subjective sensation of oral dryness may also lead to the development of depression states.^[46]^ It has been confirmed with our data which demonstrated a significant positive correlation between increased clinical symptoms of oral dryness with increased PHQ-9 scale values. Contrary to Marques-Vidal et al,^[38]^ who were determining depression among students (aged 21 ± 3) with the use of the Hospital Anxiety and Depression Scale, we did not observe a relationship between depression and toothache and gum bleeding.

Oral health can influence mental health in a similar way mental health has an impact on oral health and the results of treatment. Assessing depression and oral health condition with the use of a questionnaire examination, O’Neil at al simultaneously controlled the C-reactive protein (CRP). The researchers demonstrated a correlation between poor health condition and depression; at the same time, their study did not find inflammation (CRP) to influence the relationship between poor oral health and depression.^[13]^ Similar results were obtained by Dahl et al who demonstrated that higher levels of psychological distress can influence the oral health of the elderly independently of other factors, such as smoking and having reduced number of teeth.^[47]^

Similarly, the more severe depression is detected in a patient, the more caries decayed teeth and fewer restored teeth are observed. Furthermore, problems with chewing or difficulties with speaking cause stress, depression and sometimes even lead to suicidal thoughts.^[14]^

Based on literature, there are also biological and behavioral mechanisms which explain the correlation between depression and oral health. Increased severity of depression is linked to poorer oral hygiene which is caused by reduced salivary secretion and increased lactobacillus counts which are side effects of antidepressants.^[48,49]^ Behavioral mechanisms, in turn, result from dietary negligence of patients suffering from depression.^[50,51]^

Increased risk of dental caries is observed as a result of abandoning dental hygienic treatments, changing eating habits in favor of products rich in fermentable carbohydrates, and hyposalivation.^[52]^ Therefore, by leading to oral dryness, cariogenic diet, and increased occurrence of oral infections, physiological consequences of depression commence a cascade of changes that result in more frequent prevalence of pathological lesions in the oral cavity. The opposite results were obtained by Mendes at al who observed that presence of depression in older persons is not associated with any alteration in the normative conditions of oral health.^[53]^

Several limitations have to be considered in a discussion about the results of this study. First, this study was conducted by using self-reported questionnaire to report data such as PHQ-9, which could lead to identification bias. However, some studies showed the use of this questionnaire to be a valid and reliable method of screening for depression.

Second, the use of survey data did not allow us to explain temporal relationships or to show inferences on causality. Third, analyzing the obtained results, it is essential to bear in mind a possible self-selection error of the study participants, that is, the participants who joined the study were worried that they were having dental problems, or they were aware of their dental problems and were looking for help; also, there may have been those who refused to participate in the study due to dental fear. Moreover, another interfering factor resulted from the application of the exclusion criteria. The study was limited by the exclusion of patients with coexisting systemic diseases in which dental pocket probing leading to transient bacteremia might have posed a risk for the patient's overall health condition, which concerned many patients in this age group. Finally, excluding patients with a mental disorder might have constituted another interfering factor, since depression could also be a symptom of mental disorder.

## 5 Conclusions

The results of our study have shown that among people aged 65 and over, the severity of depression increases with a higher number of MT, the number of decayed teeth, as well as prevalence of oral dryness. Therefore, our data indicate that some oral health parameters could influence a patient's well-being. and people with depression could have impaired oral health.

## Author contributions

**Conceptualization:** Katarzyna Skośkiewicz-Malinowska, Barbara Malicka, Marek Zietek, Urszula Kaczmarek.

**Data curation:** Katarzyna Skośkiewicz-Malinowska, Barbara Malicka.

**Investigation:** Katarzyna Skośkieiwcz-Malinowska, Barbara Malicka.

**Methodology:** Katarzyna Skośkiewicz-Malinowska, Urszula Kaczmarek.

**Supervision:** Urszula Kaczmarek.

**Writing – original draft:** Katarzyna Skośkiewicz-Malinowska, Barbara Malicka, Urszula Kaczmarek.

**Writing – review & editing:** Katarzyna Skośkiewicz-Malinowska, Marek Zietek, Urszula Kaczmarek.
